# Identification of a Novel Strong and Ubiquitous Promoter/Enhancer in the Silkworm *Bombyx mori*

**DOI:** 10.1534/g3.114.011643

**Published:** 2014-05-23

**Authors:** Takuya Tsubota, Keiro Uchino, Takao K Suzuki, Hiromitsu Tanaka, Takumi Kayukawa, Tetsuro Shinoda, Hideki Sezutsu

**Affiliations:** * Transgenic Silkworm Research, National Institute of Agrobiological Sciences, 1-2 Owashi, Tsukuba, Ibaraki 305-8634, Japan; † Insect Mimetics Research, National Institute of Agrobiological Sciences, 1-2 Owashi, Tsukuba, Ibaraki 305-8634, Japan; ‡ Insect Growth Regulation Research Unit, National Institute of Agrobiological Sciences, 1-2 Owashi, Tsukuba, Ibaraki 305-8634, Japan

**Keywords:** promoter/enhancer, hsp90, silkworm, enhancer trap, transgenic

## Abstract

Transgenic techniques offer a valuable tool for determining gene functions. Although various promoters are available for use in gene overexpression, gene knockdown, and identification of transgenic individuals, there is nevertheless a lack of versatile promoters for such studies, and this dearth acts as a bottleneck, especially with regard to nonmodel organisms. Here, we succeeded in identifying a novel strong and ubiquitous promoter/enhancer in the silkworm. We identified a unique silkworm strain whose reporter gene showed strong and ubiquitous expression during the establishment of enhancer trap strains. In this strain, the transposon was inserted into the 5′UTR of *hsp90*, a housekeeping gene that is abundantly expressed in a range of tissues. To determine whether the promoter/enhancer of *hsp90* could be used to induce strong gene expression, a 2.9-kb upstream genomic fragment of *hsp90* was isolated (hsp90^P2.9k^), and its transcriptional activation activity was examined. Strikingly, hsp90^P2.9k^ induced strong gene expression in silkworm cell cultures and also strongly induced gene expression in various tissues and developmental stages of the silkworm. hsp90^P2.9k^ also exhibited significant promoter/enhancer activity in Sf9, a cell culture from the armyworm, suggesting that this fragment might possibly be used as a gene expression tool in other Lepidoptera. We further found that 2.0 kb of hsp90^P2.9k^ is sufficient for the induction of strong gene expression. We believe that this element will be of value for a range of studies such as targeted gene overexpression, gene knockdown and marker gene expression, not only in the silkworm but also in other insect species.

Transgenesis is an important research technique in both basic and applied biological sciences. With respect to basic research, transgenic manipulations offer the opportunity to carry out *in vivo* functional analysis of genes or to identify their *cis* regulatory elements. The use of transgenesis methodologies has contributed greatly to advances in a wide range of biological research, including molecular biology, genetics, physiology, and others. In applied research, transgenic organisms have been used as a bioreactor for production of medically important compounds or materials or for pest control (Robinson *et al.* 2004; Tatematsu *et al.* 2012). For both basic and applied research, it is important that expression of a transgene is regulated very precisely, both in target tissues and at the appropriate time. Because this regulation usually is mediated by the activity of the promoter connected to the transgene, it is clear that promoter activity is very critical for transgenic analyses. However, the lack of a suitable promoter is still a bottleneck for some studies, especially those using nonmodel organisms.

The silkworm, *Bombyx mori*, has a long history of use in research. *B*. *mori* belongs to the order Lepidoptera and was the first species of this order to have its genome sequenced (The International Silkworm Genome Consortium 2008). Transgenic techniques also have been developed for use in this species, enabling studies on gene overexpression, gene knockdown, and knockout, among many others (Tamura *et al.* 2000; Imamura *et al.* 2003; Dai *et al.* 2008; Takasu *et al.* 2010; Ma *et al.* 2012; Sajwan *et al.* 2013; Takasu *et al.* 2013). In *Bombyx*, several different promoters have been exploited in these studies: the *actin A3* promoter, which can induce widespread gene expression (Tamura *et al.* 2000; Imamura *et al.* 2003); the 3xP3 artificial promoter, which has three binding sites for Pax-6 and functions in the stemmata or complex eyes (Berghammer *et al.* 1999; Thomas *et al.* 2002; Imamura *et al.* 2003); the *hsp70* promoter, which can induce a significant level of gene expression after heat shock treatment (Uhlířová *et al.* 2002); and the *sericin* or *fibroin* promoter, which acts specifically in the middle or posterior silk gland (Imamura *et al.* 2003; Sezutsu *et al.* 2009; Tatematsu *et al.* 2010). In addition, we recently have succeeded in identifying a novel hemocyte oenocytoid promoter (Tsubota *et al.* 2014). These promoters have been used not only for gene function analyses or recombinant protein production but also for identification of transgenic organisms (Thomas *et al.* 2002). Much of the recent progress in transgenic silkworm research can be attributed to the exploitation of such promoters. Unfortunately, however, these promoters nevertheless have limitations regarding their application. The A3 promoter, for example, is not ubiquitous but shows little to no activity in several larval tissues and in the embryonic stage (Tamura *et al.* 2000; Uchino *et al.* 2008). It is thus not practical to use this promoter for functional analyses in these tissues and stage. Another recently described baculovirus immediate-early promoter, *ie1*, also exhibits limited activity in the larvae (Masumoto *et al.* 2012), demonstrating the difficulty of identifying a *bona fide* ubiquitous promoter. The 3xP3 promoter is widely used for transgenic marker gene expression; however, because the promoter functions just in a very limited region, screening is difficult and time-consuming (Thomas *et al.* 2002). As a consequence of the problems associated with use of many of the known promoters, identification of more versatile promoters is crucial for exploitation of transgenic methods to promote further progress in silkworm research.

The enhancer trap system provides one of the most powerful tools in functional genomic research. In this system, a transposon is induced to insert randomly into the host genome using transposase activity. Usually the transposon contains a reporter (*e.g.*, green fluorescent protein [GFP], cyan fluorescent protein [CFP], or lacZ) or a driver gene (*e.g.*, GAL4). If the transposon is inserted within the promoter/enhancer region of a gene, its activity can be detected by monitoring reporter gene expression. This method was first exploited in the fruit fly *Drosophila melanogaster* (O’Kane and Gehring 1987; Cooley *et al.* 1988) and now applied to various organisms, including insects such as red flour beetle (*Tribolium castaneum*) and mosquito (*Anopheles stephensi*), vertebrates such as zebrafish (*Danio rerio*) and medaka (*Oryzias latipes*), and plants such as rice (*Oryza sativa*) and thale cress (*Arabidopsis thaliana*) (Lorenzen *et al.* 2003; Ito *et al.* 2004; Parinov *et al.* 2004; Ellingsen *et al.* 2005; Liu *et al.* 2005; Lorenzen *et al.* 2007; O’Brochta *et al.* 2011). A significant number of genes or their promoter/enhancer elements have been identified using this system (Bier *et al.* 1989; Wilson *et al.* 1989; Ito *et al.* 2004; Parinov *et al.* 2004; Ellingsen *et al.* 2005; Liu *et al.* 2005; Vijaybhaskar *et al.* 2008). Our laboratory has previously established more than 300 silkworm enhancer trap strains using the *piggyBac* transposon and released descriptions of reporter gene expression into public databases (Uchino *et al.* 2008; Shimomura *et al.* 2009). The detection of highly variable expression profiles strongly indicates that the silkworm enhancer trap system can contribute to the identification of a wide variety of novel genes and/or their promoter/enhancer elements (Uchino *et al.* 2008). These strains are also valuable resources for tissue-specific driver strains since the transposon contains a *GAL4* gene (Ito *et al.* 2008; Daimon *et al.* 2012).

In this study, we identified a unique transgenic silkworm strain whose reporter gene showed strong and widespread expression pattern during the process of enhancer trap strain establishment. Analysis of the insertion site revealed that the transposon was inserted into *Bombyx hsp90*, a housekeeping gene that is expressed abundantly in various tissues. We examined the promoter/enhancer activity of an *hsp90* 2.9 kb upstream sequence (hsp90^P2.9k^) and found that it induced strong gene expression in silkworm cell cultures as well as in a range of tissues and developmental stages. hsp90^P2.9k^ also drove strong transcription in an armyworm cell line, Sf9, suggesting that the fragment could be used as a gene expression tool in other lepidopteran species. We further found that just 2.0 kb would be sufficient for the induction of strong gene expression. We believe that the identified fragment will be of value in various types of investigation, such as widespread gene expression or efficient transgenic individual screening, and will thereby contribute to further advances in research in silkworms and other insects.

## Materials and Methods

### Silkworm strains

Silkworms, *B. mori*, were reared on an artificial diet (Nihon Nosan Kogyo, Yokohama, Japan) at 25° under a 12-hr light/dark photoperiod. A silkworm enhancer trap strain, *AyFib-042*, was used as a mutator strain (Sezutsu *et al.* 2009). This strain was crossed with a jumpstarter strain, *Js15* (Uchino *et al.* 2008), for the mobilization of the *piggyBac* transposon. The established strains were crossed with the *UAS-GFP* homozygous strain (Uchino *et al.* 2006) and the mobilization of the transposon was confirmed by the lack of the stripe-like GFP expression of the original *AyFib-042* strain (Sezutsu *et al.* 2009). The *AyFib-431a* strain was obtained in this way.

### Detection of GFP and DsRed fluorescence

The expression of the fluorescent proteins GFP and DsRed was detected using an MZFLIII (Leica, Solms, Germany), MZ16FA (Leica), SZX16 (Olympus, Tokyo, Japan), or VB-S20 fluorescence microscope (Keyence, Osaka, Japan) with a GFP band pass filter (excitation: 440/70 nm, emission: 525/50 nm or excitation: 440/70 nm, emission: 535/550 nm or excitation: 460/80 nm, emission: 495/540 nm), GFP long-pass filter (excitation: 440/70 nm, emission: 510 nm) or DsRed filter (excitation: 525/40 nm, emission: 572 nm or excitation: 530/45 nm, emission: 620/60 nm). Images were captured using a Leica DC200 (Leica), Leica DFC300FX (Leica), DP71 (Olympus), or VB-7000 (Keyence) system.

### Inverse polymerase chain reaction (PCR)

Genomic DNA was isolated from adult moths by sodium dodecyl sulfate−phenol extraction (Ohshima and Suzuki 1977). The DNA was digested with *Nco*I and heated at 70° for 15 min to inactivate the enzymes. The digested DNA was purified using a QIAquick PCR Purification Kit (QIAGEN, Hilden, Germany) or QIAquick Gel Extraction Kit (QIAGEN) and ligated using T4 DNA ligase (New England Biolabs, Ipswich, MA) at 4°overnight. The DNA was purified using a QIAquick PCR Purification Kit (QIAGEN) and was used as the template for PCR. PCR was carried out using the ExTaq polymerase (TaKaRa, Otsu, Japan) as follows: 95° for 2 min followed by five cycles of 95° for 30 sec, 65° for 30 sec and 72° for 2 min, 5 cycles of 95° for 30 sec, 60° for 30 sec and 72° for 2 min, 35 cycles of 95° for 30 sec, 55° for 30 sec and 72° for 2 min, and 72° for 10 min. The amplified fragments were sequenced using Applied Biosystems 3130*xl* (Life Technologies, Carlsbad, CA) after cycle sequencing with BigDye Terminator V3.1 (Life Technologies). The obtained sequence was subjected to a BLAST search in the silkworm genome database, KAIKObase (http://sgp.dna.affrc.go.jp/KAIKObase/) for the identification of the insertion site.

### Southern hybridization analysis

Southern hybridization analysis was performed as described in Sezutsu *et al.* (2009). In brief, 2 µg of the genomic DNA was digested with *Nco*I or *Sac*I and subjected to electrophoresis, blotted onto a Hybond-N^+^ nylon membrane (GE Healthcare, Chalfont St. Giles, UK), hybridized with a GAL4 probe and detected using an Alkphos Direct Labeling and Detection System (GE Healthcare).

### Probe synthesis for *in situ* hybridization

5′-ATGCCGGAAGAAATGGAGAC-3′ and 5′-TGCTCGGAACTCTAACTGAC-3′ primers with T7 sequence was used for PCR amplification of the *hsp90* gene. The PCR product was purified and used as a template for a probe labeling reaction. For probe labeling, DIG RNA labeling kit (SP6/T7) was used (Roche Diagnostics, Basel, Switzerland).

### *In situ* hybridization

Embryos (ca. stage 20) were dissected and fixed in 4% paraformaldehyde at room temperature overnight. They were stored in 100% methanol at −20° until use. After replacement of the methanol with phosphate-buffered saline (PBS), the embryos were placed in 0.2 M HCl for 10 min and washed with PBS. They were then treated with 10 µg/mL proteinase K (Roche diagnostics) at 37° for 30 min, washed with PBS, and acetylated using 0.1 M triethanolamine-HCl (pH 8.0) and 0.25% acetic anhydride for 10 min. This solution was gradually replaced by hybridization buffer, and the embryos were hybridized with a labeled probe (100 ng/ml concentration) at 60° overnight. After hybridization, they were washed in PBS, and unhybridized probe was degraded using 50 µg/mL RNaseA. For antibody reaction, the blocking reaction was carried out using 10% Goat Normal Serum, and the anti-Dig antibody (Roche Diagnostics) was applied at a 1:2000 dilution. The embryos were then stained with NBT/BCIP (Roche Diagnostics), mounted in 100% glycerol and examined using an Axioplan microscope (Carl Zeiss, Oberkochen, Germany).

### Reverse-transcription PCR of hsp90

Total RNAs were extracted from each tissue of *w-c* spinning stage larvae using ISOGEN (Nippongene, Tokyo, Japan) and SV Total RNA Isolation System (Promega, Madison, WI). The RNA concentration was measured using a NanoDrop ND2000 UV spectrophotometer (Thermo Fisher Scientific Inc., Waltham, MA). The RNA was used for reverse-transcription using Superscript III (Life Technologies). The KOD-FX polymerase (Toyobo, Osaka, Japan) was used for PCR, with the following primer sequences: 5′-ATGCCGGAAGAAATGGAGAC-3′ and 5′-TGCTCGGAACTCTAACTGAC-3′ for *hsp90* and 5′-CAGGCGGTTCAAGGGTCAATAC-3′ and 5′-TGCTGGGCTCTTTCCACGA-3′ for *rp49*.

### Plasmid construction for the luciferase reporter assay and for transgenic silkworm generation

The pGL3-A3 plasmid was generated for the luciferase reporter assay of the A3 promoter. First, the 682 bp A3 promoter fragment was amplified from pHA3PIG (Tamura *et al.* 2000) using primers 5′-CTAGCTAGCCCGGGCTCAAGCTTGATGCG-3′ and 5′-CCGCTCGAGTGAATTAGTCTGCAAGAA-3′. The amplified fragment was inserted into the *Nhe* I/*Xho* I site of the pGL3 vector (Promega) to generate pGL3-A3. A pGL3- hsp90^P2.9k^ plasmid was generated for the hsp90^P2.9k^ luciferase assay. The hsp90^P2.9k^ genomic sequence was first determined by inverse PCR because the genome sequence upstream of *hsp90* was not available in the KAIKObase. After sequence determination, the hsp90^P2.9k^ genomic fragment was amplified from the *w-c* strain using primers 5′-TTAATTCACACAAAATGACTAGAGGG-3′ and 5′-CCATGGCTCAGTTCGCTTTAAATA-3′ and was inserted into pCR-BluntII-TOPO vector (Life Technologies). We verified the sequence, and the hsp90^P2.9k^ fragment was inserted into the *Nhe* I/*Xho* I site of the pGL3 vector to generate pGL3-hsp90^P2.9k^. pGL3-hsp90^P2.0k^ or pGL3-hsp90^P1.6k^ plasmid was generated for the luciferase reporter assay of hsp90^P2.9k^ partial fragment. The 916- to 2033-bp (hsp90^P2.0k^) or 1330- to 2033-bp (hsp90^P1.6k^) fragment of hsp90^P2.9k^ was first amplified from pGL3-hsp90^P2.9k^ using primers 5′-TCGCGTTTCCTTCACTCGCG-3′ and 5′-TCTAGAAAAGCATCGAAATTTTAACATTACAGA-3′ or 5′-GGCGCTTCTCAAATGGAACTTTCG-3′ and 5′- TCTAGAAAAGCATCGAAATTTTAACATTACAGA-3′, respectively. The amplified fragment was inserted into the pCR-BluntII-TOPO vector and the sequence was verified. Next, the plasmid was digested with *Xba*I, and the nucleotides downstream of hsp90^P2.9k^ 2034 bp was connected by inserting the fragment obtained by *Xba*I digestion of pGL3-hsp90^P2.9k^. The plasmid (pCR-BluntII-hsp90^P2.0k^ or pCR-BluntII-hsp90^P1.6k^) was digested with *Nhe*I/*Xho*I, and the hsp90^P2.0k^ or hsp90^P1.6k^ fragment was inserted into the *Nhe*I/*Xho*I site of pGL3 to generate pGL3-hsp90^P2.0k^ or pGL3-hsp90^P1.6k^. The *hsp90-GFP* transgenic strain was generated using the pBachsp90GFP-3xP3DsRed plasmid. To construct this vector, the pBacA3dGAL4/3xP3-DsRed(ANB) vector (Tsubota *et al.* 2014) was digested with *Asc*I/*Xba*I and the A3dGAL4 fragment was removed. Then, GFP and SV40 terminator sequences were inserted to generate pBacGFP-3xP3DsRed. Next, the hsp90^P2.9k^ fragment was inserted into the *Xba* I site located upstream of GFP to generate pBachsp90GFP-3xP3DsRed.

### Luciferase reporter assay

The luciferase reporter assay using NIAS-Bm-oyanagi2 was conducted as described in Tanaka *et al.* (2009). In summary, the reporter plasmid (pGL3-A3, pGL3-hsp90^P2.9k^, pGL3-hsp90^P2.0k^ or pGL3-hsp90^P1.6k^) and pOpIE2-core-Rluc (Tanaka *et al.* 2012) were transfected into NIAS-Bm-oyanagi2 cells using FuGENE HD (Promega). Firefly luciferase activity in cell extracts was measured 72 hr later and normalized against *Renilla* luciferase activity. Both luciferase activities were measured using a lumicounter (Nition, Funabashi, Japan) with the Dual-luciferase assay system (Promega).

For the reporter assay of the cell lines NIAS-Bm-aff3 (Kayukawa *et al.* 2012), BmN (Katakura Industries Co., Tokyo, Japan), Sf9 (Tsubota *et al.* 2010), S2 (Kayukawa *et al.* 2012), or Tc81 (Kayukawa *et al.* 2013), cells were seeded at a density of 1.5 × 10^5^ cells per well in 200 µL of medium in a 96-well plate 1 d before transfection. The reporter plasmid was transfected with pIZT_RLuc vector using FuGENE HD Transfast transfection Reagent (Promega); 72 hr later, luciferase activity was measured using a luminometer ARVO (PerkinElmer, Waltham, MA) with the Dual-luciferase assay system (Promega).

### Generation of transgenic silkworms

The transgenic silkworm strain was produced by germline transformation as described elsewhere (Tamura *et al.* 2000, 2007). The plasmid DNA for the injection was purified using a QIAGEN Plasmid Midi Kit (QIAGEN). The DNA was injected into *w1-pnd* embryos at the preblastodermal stage. Screening of G1 embryos were performed around stage 25 (6 or 7 d after oviposition). The transgenic strains were maintained by crossing with diapausing *w-c* strain.

## Results

### Isolation of a novel transgenic silkworm strain with strong and ubiquitous DsRed expression

**Figure 1:**
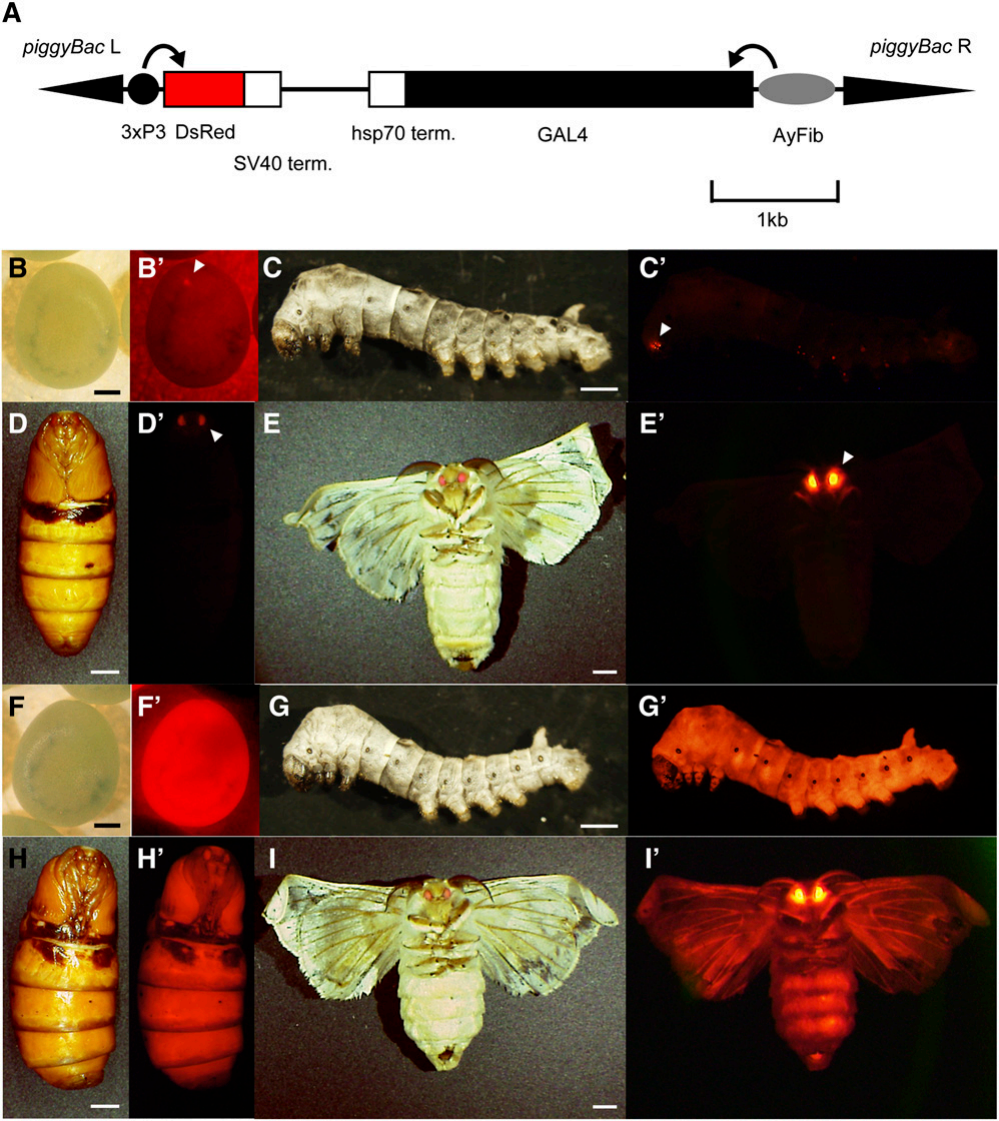
DsRed expression in *AyFib-042* and *AyFib-431*a strains. (A) Structure of the *piggyBac* transposon vector used for AyFib enhancer trap strains. L, left arm; R, right arm; 3xP3, artificial 3xP3 promoter (Berghammer *et al.* 1999); SV40 term., SV40 terminator; hsp70 term., hsp70 terminator; AyFib, *Antheraea yamamai* fibroin promoter (Sezutsu *et al.* 2009). (B−E′) Expression of DsRed protein in the *AyFib-042* strain. (B, C, D, E) Bright-field images. (B′, C′, D′, E′) DsRed-fluorescence images. (B, B′) Embryo 7 d after oviposition. (C, C′) Third instar larva. (D, D′) Pupa. (E, E′) Adult. Arrowheads in (B′), (C′), (D′), and (E′) indicate the eye-specific DsRed expression in *AyFib-042*. (F−I′) Expression of DsRed protein in *AyFib-431a* strain. (F, G, H, I) Bright-field images. (F′, G′, H′, I′) DsRed fluorescence images. (F, F′) Embryo 7 d after oviposition. (G, G′) Third instar larva. (H, H′) Pupa. (I, I′) Adult. Bars in (B, F) are 0.3 mm, (C, G) are 1 mm, and (D, E, H, I) are 3 mm.

We previously constructed a large number of silkworm enhancer trap strains for use in *Bombyx* functional genomics (Uchino *et al.* 2008). We also have proceeded to establish the novel enhancer trap strains using the *AyFib-042* strain as the mutator (see the section *Materials and Methods*), and during this process, we could obtain a unique strain designated *AyFib-431a*. *AyFib* strains possess an *Antheraea yamamai* fibroin promoter-GAL4 cassette and a 3xP3 promoter-DsRed transgenic marker (Figure 1A). The eye-specific activity of the 3xP3 promoter drives DsRed expression only in the stemmata and complex eyes (Figure 1, B−E′). In *AyFib-431a*, however, DsRed expression was strong and widespread in embryonic, larval, pupal, and adult stages (Figure 1, F−I′). The results indicated that the 3xP3 had trapped a promoter/enhancer with strong and ubiquitous activity.

To confirm that the transposon had altered its position, a southern hybridization analysis was carried out. Genomic DNAs were extracted from the *AyFib-042* and *AyFib-431a* strains and used for hybridization with a probe for the GAL4 fragment. The two strains showed different band patterns on gels (Supporting Information, Figure S1). Therefore, we conclude that the *piggyBac* transposon had indeed been mobilized and inserted into a novel genomic locus in the *AyFib-431a* strain. The southern hybridization analysis also showed that both *AyFib-042* and *AyFib-431a* have a single copy of the *piggyBac* transposon (Figure S1). Our findings suggested that the strong DsRed expression in *AyFib-431a* was mediated by the activity of a strong promoter/enhancer located at a single genomic locus.

### Detailed expression analysis of DsRed in AyFib-431a

**Figure 2:**
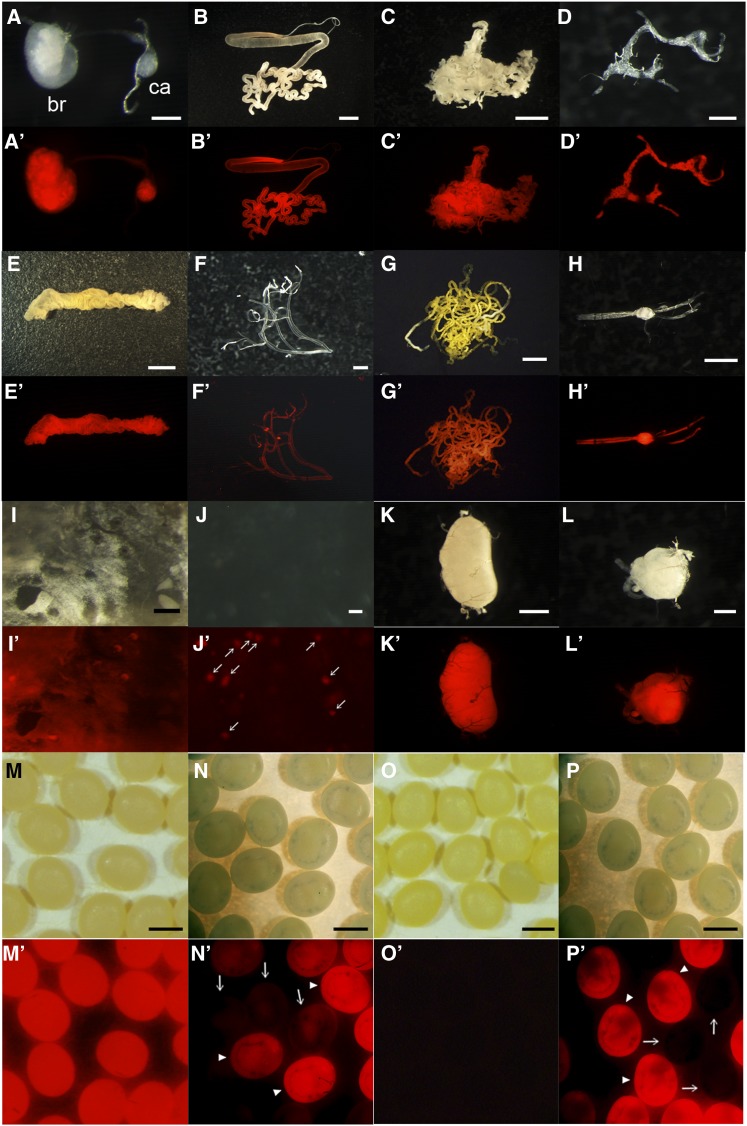
DsRed expression of *AyFib-431a* in each tissue of final instar larvae (A−L′) or in the embryo (M−P′). (A, A′) Brain and corpora allata. (B, B′) Silk gland. (C, C′) Fat body. (D, D′) Prothoracic gland. (E, E′) Gut. (F, F′) Trachea. (G, G′) Malpighian tubules. (H, H′) Ventral nerves. (I, I′) Epidermis. (J, J′) Hemolymph. (K, K′) Testis. (L, L′) Ovary. (A, B, C, D, E, F, G, H, I, J, K, L) Bright-field images. (A′, B′, C′, D′, E′, F′, G′, H′, I′, J′, K′, L′) DsRed-fluorescent images. br, brain; ca, corpora allata. Arrows in (J′) indicate hemocyte cells. (M, M′, N, N′) Eggs laid by *AyFib-431a* heterozygous female crossed with *w-c* male. (M, M′) Just after oviposition. (N, N′) 7 days after oviposition. Arrowheads indicate the putative DsRed^+^ (*AyFib-431a*/+) and arrows indicate DsRed^−^ (+/+) individuals. Residual maternal DsRed protein was detected in the putative +/+ embryos. (O, O′, P, P′) Eggs laid by *w-c* female crossed with *AyFib-431a* heterozygous male. (O, O′) Just after oviposition. (P, P′) 7 days after oviposition. Arrowheads indicate the putative DsRed^+^ (*AyFib-431a*/+) and arrows indicate DsRed^−^ (+/+) individuals. Bars represent 0.25 mm in (A, I), 5 mm in (B, E), 2.5 mm in (C, G), 0.5 mm in (D, L), 1 mm in (F, H, K, M−P), and 0.05 mm in (J).

Next, we examined DsRed expression in more detail. The expression was investigated in each larval tissues and interestingly, all of the investigated tissues, namely, brain, corpora allata, silk gland, fat body, prothoracic gland, gut, trachea, Malpighian tubules, ventral nerve, epidermis, hemocyte, testis, and ovary, exhibited strong level of DsRed expression (Figure 2, A−L′). Thus, it is suggested that the genomic region in which the *piggyBac* is inserted in *AyFib-431a* is under the regulation of a highly ubiquitous promoter/enhancer. We found that the DsRed expression levels were particularly high in the brain, corpora allata, fat body, ventral nerve, testis and ovary (Figure 2, A−L′).

The strong DsRed expression in the ovary was of interest as it suggested the possibility of maternal inheritance. To investigate this possibility, we crossed a female of *AyFib-431a* heterozygote with a male of the nontransgenic strain *w-c* and analyzed DsRed expression in the progeny. If the DsRed shows Mendelian inheritance, then the ratio of DsRed positive to negative eggs will be 1:1. Contrary to this expectation, however, all of the eggs were found to express DsRed very strongly just after oviposition (Figure 2, M and M′), suggesting that maternal inheritance has occurred. The level of DsRed expression diminished in about half of the embryos by the late embryonic stage, *i.e.*, around seven days after egg laying (Figure 2, N and N′). On the other hand, none of the progenies of the cross between *w-c* females and *AyFib-431a* heterozygous males showed DsRed immediately after oviposition, but approximately half of them showed expression of DsRed by seven days after oviposition (Figure 2, O−P′, data not shown).

### *A. yamamai* fibroin promoter activity in AyFib-431a

**Figure 3:**
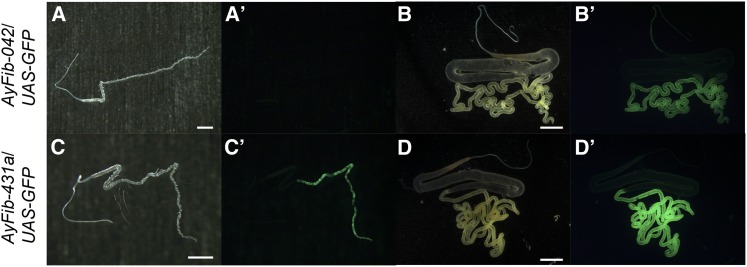
GAL4 expression in *AyFib-042* (A−B′) and *AyFib-431a* (C−D′). AyFib strains were crossed with the *UAS-GFP* homozygous strain (Uchino *et al.* 2006) and green fluorescent protein in the F1 hybrid was observed to monitor GAL4 expression. (A, A′, C, C′) First day of third instar larva. (B, B′, D, D′) Spinning stage of final instar larva. (A, B, C, D) Bright-field images. (A′, B′, C′, D′) GFP-fluorescent images. Bars represent 1 mm in (A, C) and 5 mm in (B, D).

Since *AyFib-431a* possesses not only the 3xP3 promoter but also the *A. yamamai* fibroin promoter (Figure 1A, Sezutsu *et al.* 2009), it was possible that the fibroin promoter also was activated ubiquitously, similarly to 3xP3. The *A*. *yamamai* fibroin promoter has been shown to be active just in the posterior silk gland (Figure 3, B and B′, Sezutsu *et al.* 2009). We examined fibroin promoter activity of *AyFib-431a* by observing GAL4 expression and found that the activity was still confined to the posterior silk gland (Figure 3, D and D′). Therefore, the *A*. *yamamai* fibroin did not appear to have trapped the ubiquitous promoter/enhancer that was trapped by 3xP3. Interestingly, we found a slight modification of GAL4 expression pattern. In *AyFib-042*, GAL4 expression can be detected only after the middle or late stage of fifth instar larvae (Figure 3, A and B′, Sezutsu *et al.* 2009), whereas in *AyFib-431a* its expression was detected at an earlier stage, namely, in the third instar larvae (Figure 3, C and C′) and, occasionally, even in the first instar larvae (data not shown).

### Identification of the AyFib-431a piggyBac insertion site

**Figure 4:**
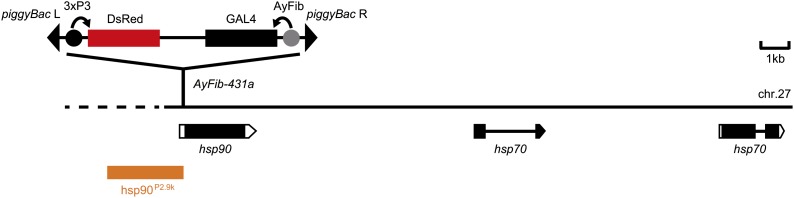
Insertion site of the *piggyBac* transposon in the *AyFib-431a* strain. The transposon is inserted into the 5′UTR of *hsp90* gene. The dashed line indicates that the genome sequence of this region is not available in the KAIKOBase.

The aforementioned results indicate that the 3xP3 but not the *A*. *yamamai* fibroin of *AyFib-431a* trapped a strong and ubiquitous silkworm promoter/enhancer. To identify the genomic region responsible for this strong and ubiquitous promoter/enhancer activity, we analyzed the *piggyBac* insertion site (see the *Materials and Methods*). This analysis indicated that the transposon was inserted into the 5′UTR of the *Bombyx hsp90* gene (Figure 4, Landais *et al.* 2001).

*hsp90* is a molecular chaperone gene that is conserved from bacteria to eukaryotes (Lindquist and Craig 1988). The gene is expressed strongly and ubiquitously in various organisms, including lepidopteran insects (Sonoda *et al.* 2006a,b, 2007; Tungjitwitayakul *et al.* 2008; Gkouvitsas *et al.* 2009; Zhang and Denlinger 2010; Xu *et al.* 2011). A previous analysis showed that silkworm Hsp90 protein is present in all larval stages (Sosalegowda *et al.* 2010). We carried out a detailed expression analysis and identified strong and widespread expression in the embryonic stage and in final instar larvae (Figure S2).

The insertion of *piggyBac* into the 5′UTR of *hsp90* suggested that the *AyFib-431a* strain might lack its function. To determine whether this was the case, we crossed *AyFib-431a* heterozygotes and examined their F1 progeny. Approximately one quarter of the progeny showed lethality at the embryonic stage (data not shown), supporting this hypothesis.

### hsp90 promoter/enhancer activity in the silkworm

**Figure 5:**
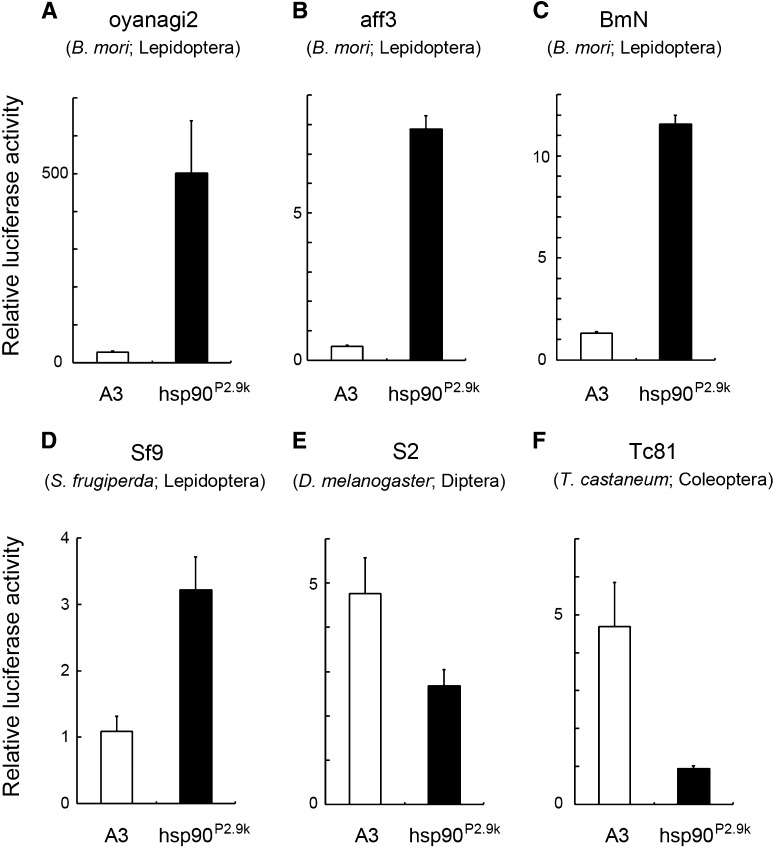
Transcriptional activation activity of *Bombyx* A3 and hsp90^P2.9k^ in cell culture. The promoter was connected to the firefly luciferase gene, and the plasmid was transfected into the following cell lines to measure activity: (A) oyanagi2, (B) aff3, (C) BmN, (D) Sf9, (E) S2, and (F) Tc81.

As *piggyBac* was inserted into the *hsp90*, a gene with a significant level of expression in various developmental stages and/or tissues (Figure S2, Sosalegowda *et al.* 2010), we hypothesized that the 3xP3 of *AyFib-431a* had trapped the *hsp90* promoter/enhancer. We therefore attempted to identify the genomic region responsible for the strong expression of *hsp90*. In mammals and flies, *hsp90* expression is regulated by a promoter/enhancer located in an upstream and/or intronic region (Xiao and Lis 1989; Shen *et al.* 1997; Zhang *et al.* 1999; Theodoraki *et al.* 2008). Because the silkworm *hsp90* lacks intron (Figure 4, Landais *et al.* 2001), we focused on its 5′ flanking region. A 2.9-kb upstream genomic fragment of *hsp90* (hsp90^P2.9k^) was amplified by PCR (Figure 4, see the section *Materials and Methods*) and its promoter/enhancer activity was examined via a luciferase reporter assay using various silkworm cell cultures. Strikingly, this fragment induced a high level of reporter gene expression in all of the *Bombyx* cells examined (Figure 5, A−C). In comparison to the A3 promoter, hsp90^P2.9k^ showed 10 times or greater activity in various cultures (Figure 5, A−C).

**Figure 6:**
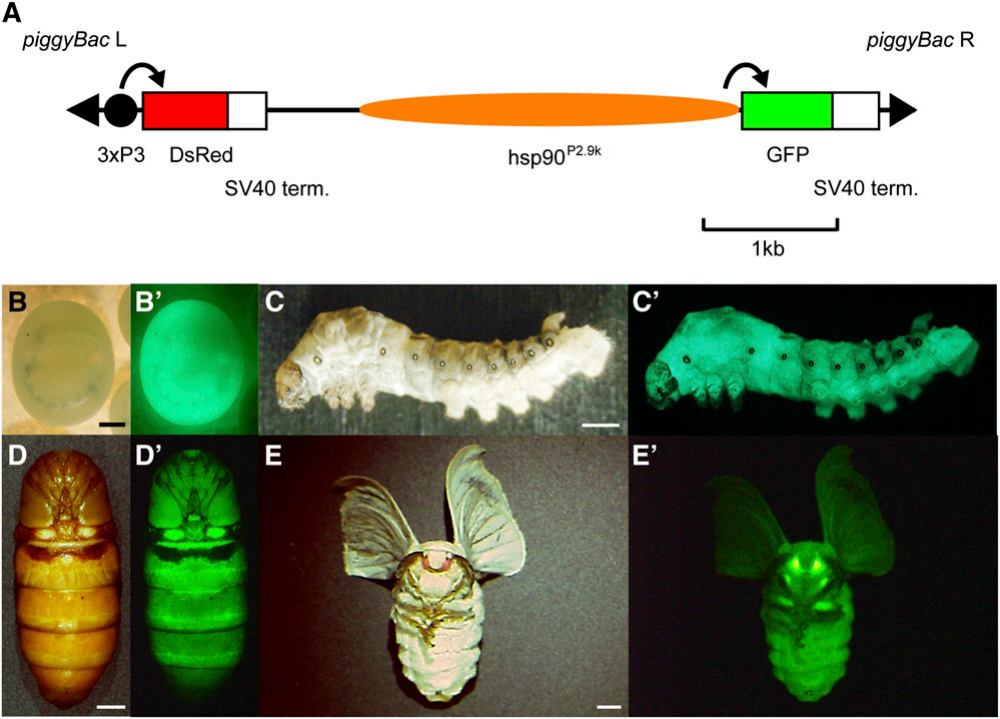
(A) Schematics of the vector used for transgenesis of *hsp90-GFP*. (B-E′) GFP expression in the *hsp90-GFP* strain. (B, C, D, E) Bright-field images. (B′, C′, D′, E′) GFP-fluorescent images. (B, B′) Embryo 7 d after oviposition. (C, C′) Third instar larva. (D, D′) Pupa. (E, E′) Adult. Bars represent 0.3 mm in (B), 1 mm in (C), and 3 mm in (D, E).

**Figure 7:**
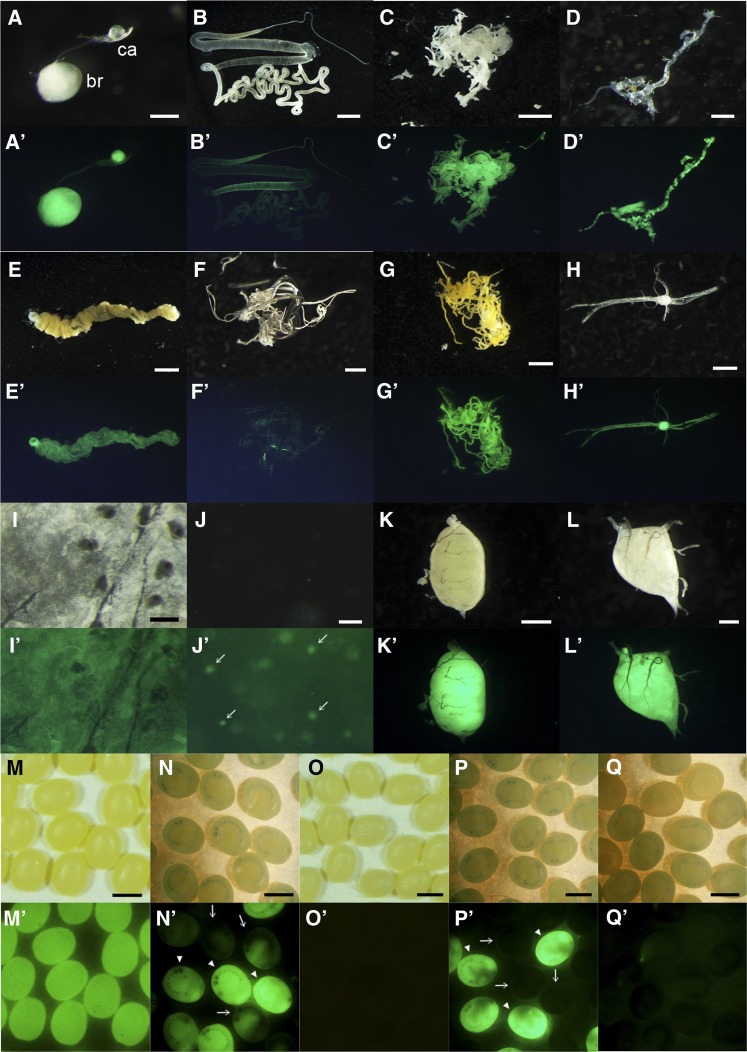
GFP expression of *hsp90-GFP* in each tissue of final instar larvae (A−L′) or in the embryo (M−P′). (A, A′) Brain and corpora allata. (B, B′) Silk gland. (C, C′) Fat body. (D, D′) Prothoracic gland. (E, E′) Gut. (F, F′) Trachea. (G, G′) Malpighian tubules. (H, H′) Ventral nerves. (I, I′) Epidermis. (J, J′) Hemolymph. (K, K′) Testis. (L, L′) Ovary. (A, B, C, D, E, F, G, H, I, J, K, L) Bright-field images. (A′, B′, C′, D′, E′, F′, G′, H′, I′, J′, K′, L′) Green fluorescent protein (GFP) images. br, brain; ca, corpora allata. Arrows in (J′) indicate hemocyte cells. (M−N′) Eggs laid by *hsp90-GFP* heterozygous female crossed with *w-c* male. (M, M′) Just after oviposition. (N, N′’) 7 d after oviposition. Arrowheads indicate the putative GFP^+^ (*hsp90-GFP*/+), and arrows indicate GFP^-^ (+/+) individuals. Residual maternal GFP protein can be detected in the putative +/+ embryos. (O−P′) Eggs laid by *w-c* female crossed with *hsp90-GFP* heterozygous male. (O, O′) Just after oviposition. (P, P′) 7 days after oviposition. Arrowheads indicate the putative GFP^+^ (*hsp90-GFP*/+) and arrows indicate GFP^−^ (+/+) individuals. (Q, Q′) GFP expression in the day seven embryo of *A3-GFP*/+ individuals. (Q) Bright-field image. (Q′) GFP-fluorescent image. Note that (N′), (P′), and (Q′) are imaged under the same exposure conditions. Bars represent 0.25 mm in (A, I), 5 mm in (B, E), 2.5 mm in (C, G), 0.5 mm in (D, L), 1 mm in (F, H, K, M−Q), and 0.05 mm in (J).

We next examined the *in vivo* activity of hsp90^P2.9k^. For this assay, a transgenic silkworm expressing GFP under hsp90^P2.9k^ was generated (*hsp90-GFP* strain, Figure 6A). We found that hsp90^P2.9k^-mediated GFP expression was present in all developmental stages (Figure 6, B−E′) and also in the various larval tissues in which we identified DsRed expression in *AyFib-431a* (Figure 7, A−L′). Interestingly, tissues with strong DsRed expression in *AyFib-431a* also expressed a high level of GFP in the *hsp90-GFP* strain (Figure 7, A−L′). The strong GFP expression in the ovary resulted in maternal inheritance similar to that seen for DsRed in the *AyFib-431a* strain (Figure 7, M−P′, compare with Figure 2, M−P′). Strong GFP expression in embryos was confirmed in four other independent *hsp90-GFP* strains (Figure S3), suggesting that the GFP expression was not due to a positional effect but was the result of hsp90^P2.9k^ activity. We also compared the levels of GFP expression in *hsp90-GFP* and *A3-GFP* strains and found a considerably higher level of expression in the former (Figure 7, Q and Q′). Taken together, our findings indicate that the hsp90^P2.9k^ fragment is responsible for the high level of expression of the *Bombyx hsp90* gene *in vivo* and also suggest that the strong and ubiquitous DsRed expression of *AyFib-431a* was, in part at least, due to the activity of hsp90^P2.9k^.

### hsp90^P2.9k^ activity in cells of other insect species

We investigated whether the hsp90^P2.9k^ fragment could also be used as a gene expression tool for other insect species. In Sf9 cells, which are derived from the lepidopteran *Spodoptera frugiperda*, hsp90^P2.9k^ induced stronger gene expression than *Bombyx* A3 by a factor of more than three (Figure 5D). A previous analysis showed that *Bombyx* A3 exhibited substantial promoter activity in the larvae of the swallowtail butterfly *Papilio xuthus* (Ando and Fujiwara 2013). Therefore, we conclude that hsp90^P2.9k^ can act as a strong and/or ubiquitous promoter/enhancer in Lepidoptera. However, hsp90^P2.9k^ showed weaker activity than *Bombyx* A3 in the fruit fly and red flour beetle cells (Figure 5, E and F). It would therefore be necessary to improve its function in order to use this fragment as a significant gene expression tool in non-Lepidopteran insects.

### The promoter/enhancer activity of hsp90^P2.9k^ partial fragment

**Figure 8:**
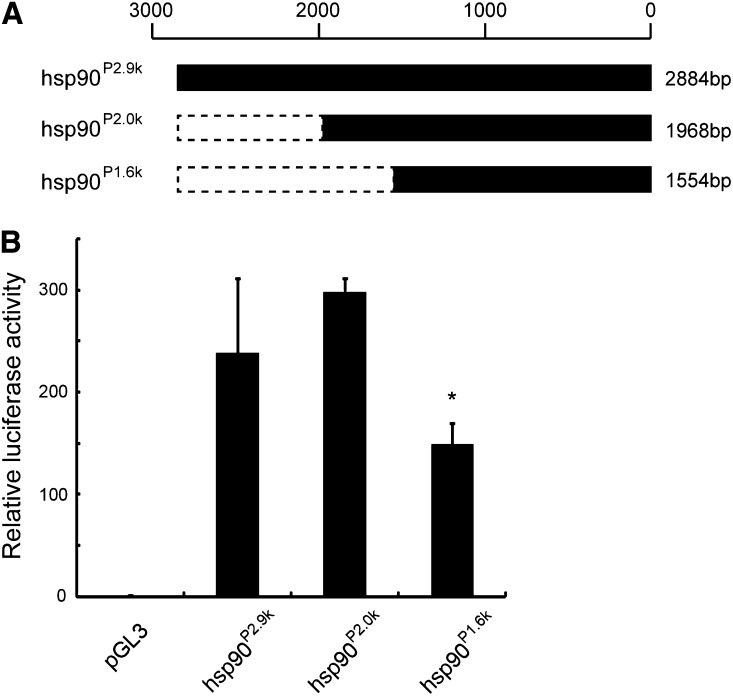
Promoter activity of hsp90^P2.9k^ and its partial fragment. (A) Schematics of the promoter fragment used in this analysis. The name of each fragment is shown to the left, and the nucleotide length is shown to the right. (B) The transcription activation activity of each fragment. The promoter was connected to the firefly luciferase gene, and the plasmid was transfected into the oyanagi2 cells to measure activity. For pGL3, just the empty vector was transfected. *P < 0.05 *vs.* hsp90^P2.9k^ (Welch’s *t*-test).

As shown above, we revealed that hsp90^P2.9k^ fragment can be used as a valuable tool for various research fields in the silkworm and other related species. However, this fragment is 2.9 kb in length; it would be more preferable to minimize this fragment for use. Thus, we deleted the fragment and investigated whether the strong promoter/enhancer activity is still present. In case that the 5′ ∼900 bp was removed (hsp90^P2.0k^), its transcription activation activity was almost comparable to that of the full-length (Figure 8). Therefore, this 2.0-kb fragment can potentially be used as a tool for strong gene expression instead of hsp90^P2.9k^. However, the removal of additional ∼400-bp sequence (hsp90^P1.6k^) resulted in the significant reduction of the activity (Figure 8). We conclude that the hsp90^P2.0k^ would be the current minimum fragment that is applicable to the induction of strong gene expression.

## Discussion

Recent progress in transgenic techniques has provided the means to perform a wide range of gene functional analyses. These techniques have now been applied to a range of insect species, such as *D*. *melanogaster* (Diptera), *Ceratitis capitata* (Diptera), *Anopheles gambiae* (Diptera), *A. stephensi* (Diptera), *B. mori* (Lepidoptera), *Plutella xylostella* (Lepidoptera), *Cydia pomonella* (Lepidoptera), *T*. *castaneum* (Coleoptera), *Athalia rosae* (Hymenoptera), and *Gryllus bimaculatus* (Orthoptera) (Rubin and Spradling 1982; Miller *et al.* 1987; Catteruccia *et al.* 2000; Tamura *et al.* 2000; Lorenzen *et al.* 2003; Sumitani *et al.* 2003; Nakamura *et al.* 2010; Ferguson *et al.* 2011; Martins *et al.* 2012). However, at present, a restricted set of promoters are available for functional studies except for *D*. *melanogaster*. In the silkworm, for example, the A3 and ie1 promoters show limited activity in some tissues and/or developmental stages (Uchino *et al.* 2008; Masumoto *et al.* 2012). This restricts the use of these promoters as a tool for strong and widespread gene expression. The A3 promoter has another drawback in that it is very susceptible to positional effects (Uchino *et al.* 2006, 2008). The promoter identified here, hsp90^P2.9k^, offers the possibility of strong gene expression across many cell and tissue types and in different developmental stages. Its ability to induce gene expression in a wide variety of larval tissues should be of especial value because this will provide greater opportunity for *in vivo* gene function analyses. Use of hsp90^P2.9k^ will enable functional studies in tissues such as the ovary and testis in which no active promoters have been available to date. Its activity in the ovary and the maternal transmission of transcripts and/or proteins will be of value to the analysis of early embryogenesis. Similar maternal promoters were previously not available in insects except for *D*. *melanogaster* (Rørth 1998). We also showed that hsp90^P2.9k^ is less sensitive to the positional effects (Figure S3). In addition to functional analyses, hsp90^P2.9k^ could be used for transgenic marker gene expression. Because this fragment induced very strong and ubiquitous gene expression in embryos (Figure 6B′), its employment would make screening much more efficient. It should also be possible to use hsp90^P2.9k^ for more effective production of the recombinant proteins, such as drugs or other materials, through transgene expression. Taken together, exploitation of this novel promoter/enhancer might be expected to benefit research across both basic and practical sciences. In an attempt to minimize this fragment, we found that ∼2.0 kb of hsp90^P2.9k^ would be sufficient for the induction of strong gene expression (Figure 8). We are now in the process for further determining the core element of this fragment.

The hsp90^P2.9k^ promoter/enhancer identified here is also of relevance to studies in species other than the silkworm. As described previously, the *in vivo* functional analysis is now possible in a number of insect species. In addition, such analysis should also be feasible even in nonmodel organisms because of the recent development of a method for rapid and efficient gene introduction, namely, electroporation-mediated somatic transgenesis, (Ando and Fujiwara 2013). The development and exploitation of versatile and/or universal promoters is thus a topic of considerable interest. In this context, our result that a novel strong and ubiquitous promoter was identified in the silkworm, a non-Drosophilid insect, should have a significant implication. This promoter/enhancer can be applicable to multiple purposes in the silkworm research, as described. Moreover, we consider that hsp90^P2.9k^ showed clear evidence of being use in studies of other lepidopteran insects because: 1) it exhibited more activity than A3 promoter in both silkworm and armyworm cell cultures (Figure 5, A−D); 2) *Bombyx* A3 has been shown to be active in the swallowtail butterfly (Ando and Fujiwara 2013); and 3) *hsp90* is expressed strongly and ubiquitously in various Lepidoptera (Sonoda *et al.* 2006a,b, 2007; Tungjitwitayakul *et al.* 2008; Gkouvitsas *et al.* 2009; Zhang and Denlinger 2010; Xu *et al.* 2011). The widespread activity of hsp90^P2.9k^ in the silkworm indicates that this fragment is also likely to be ubiquitously active in other lepidopteran species. However, use of *Bombyx* hsp90^P2.9k^ in non-Lepidopteran insects might be more problematic because it showed weak activity in *Drosophila* and *Tribolium* cells (Figure 5, E and F). Weak expression driven by a *Bombyx* enhancer in distantly related insect species could be due to evolutionary divergence of the core transcription machinery, as has been suggested recently for *T*. *castaneum* (Schinko *et al.* 2010). To more generally test the capacity of *hsp90* promoters to drive ubiquitous expression, it might be therefore necessary to isolate the respective endogenous promoters in the species of interest.

The regulation of *hsp90* gene transcription has been studied previously in the yeast, fungus, fruit fly, medfly, mouse, and humans (Xiao and Lis 1989; Giardina and Lis 1995; Dale *et al.* 1997; Shen *et al.* 1997; Erkine *et al.* 1999; Zhang *et al.* 1999; Theodoraki *et al.* 2008; Lamoth *et al.* 2014). These studies have reported that transcription of the gene is activated by a transcription factor, heat shock factor (HSF), that binds to the *cis*-element of *hsp90* [heat shock element (HSE)] (Giardina and Lis 1995; Shen *et al.* 1997; Erkine *et al.* 1999). HSF also regulates the expression of other genes, such as *hsp70*, via HSEs whose consensus sequences are identical to that of *hsp90* (Wu 1984). Studies in the silkworm have shown that HSF can bind to the HSE of *hsp70* or *samui* genes, suggesting the possible conservation of HSF function in this species (Kihara *et al.* 2011). We analyzed *Bombyx* hsp90^P2.9k^ or hsp90^P2.0k^ and found several HSE-like sequences (Figure S4). Thus, it is very likely that these elements play critical roles for *hsp90* transcription. Previously, we found that a 108-bp upstream sequence of *Bombyx hsp90* that does not contain any HSEs shows no promoter activity (Figure S4, Uchino *et al.* 2006); this observation is suggestive of a possible role for HSE-like sequences. Interestingly, a search of lepidopteran genomes showed that these elements are also present upstream of *hsp90* gene in species such as *Manduca sexta*, *Danaus plex*, and *Heliconius melpomene* (data not shown). Apart from HSEs, very little is known about *cis* regulatory elements for *hsp90*. In *D*. *melanogaster* it has been suggested that a 7 bp module (CGTTTTG) might play a role in expression of *hsp90* in the ovary (Xiao and Lis 1989), but this element does not appear to be present in the medfly (Theodoraki *et al.* 2008). The silkworm likewise does not have this element despite the fact that *Bombyx* hsp90^P2.9k^ can induce strong gene expression in the ovary (Figure 7L′, data not shown). One possibility is that the strong *hsp90* expression in the ovary might be mediated by species-specific mechanisms or strategies.

This study has shown that a *Bombyx hsp90* upstream fragment can be used as an effective gene expression tool in both *Bombyx* and closely related species. Our analyses also demonstrated that use of this fragment was effectively limited to Lepidoptera. We are currently attempting to identify further versatile promoters that can be used in a wide range of organisms. Exploitation of emerging genomic information and/or gene expression data, in combination with a variety of *in vivo* analytical tools, should allow us to identify further useful promoters.

## Supplementary Material

Supporting Information
