# Supplementary material for: Identification of a Novel Strong and Ubiquitous Promoter/Enhancer in the Silkworm Bombyx mori
Source: G3 (Bethesda). 2014 May 23;4(7):1347–57. doi: 10.1534/g3.114.011643 (PMC4455783; doi:10.1534/g3.114.011643)
Supplement: Supporting Information [file supp_4_7_1347__index.html]

Identification of a Novel Strong and Ubiquitous Promoter/Enhancer in the Silkworm Bombyx mori — Supporting Information 

# Identification of a Novel Strong and Ubiquitous Promoter/Enhancer in the Silkworm *Bombyx mori*

## Supporting Information for Tsubota *et al.*, 2014

**Files in this Data Supplement:**

- Supporting Information - Figures S1-S4 (PDF, 538 KB)
- Figure S1 - Southern hybridyzation of *AyFib-042* and *AyFib-431a* genomic DNAs. (PDF, 151 KB)
- Figure S2 - Expression of *hsp90*. (PDF, 379 KB)
- Figure S3 - Embryonic GFP expression in four additional *hsp90-GFP* transgenic strains. (PDF, 395 KB)
- Figure S4 - Sequence of *Bombyx* hsp90P2.9k fragment. (PDF, 702 KB)
