# Supplementary material for: Identification of a Novel Strong and Ubiquitous Promoter/Enhancer in the Silkworm Bombyx mori
Source: G3 (Bethesda). 2014 May 23;4(7):1347–57. doi: 10.1534/g3.114.011643 (PMC4455783; doi:10.1534/g3.114.011643)
Supplement: Supporting Information [file supp_g3.114.011643_011643SI.pdf]

G3·Genes | Genomes | Genetics

Identification of a novel strong and ubiquitous promoter/enhancer in the silkworm *Bombyx mori*

Takuya Tsubota[*,1], Keiro Uchino[*,1], Takao K Suzuki[*], Hiromitsu Tanaka[§], Takumi Kayukawa[†], Tetsuro Shinoda[†], Hideki Sezutsu[*,2]

[*]Transgenic Silkworm Research, [§]Insect Mimetics Research and [†]Insect Growth Regulation Research Unit, National Institute of Agrobiological Sciences, 1-2 Owashi, Tsukuba, Ibaraki 305-8634, Japan

[1] These authors contributed equally to this work.
[2] Corresponding author.
Hideki Sezutsu
Transgenic Silkworm Research Unit,
National Institute of Agrobiological Sciences,
1-2 Owashi, Tsukuba, Ibaraki 305-8634, Japan
Tel: +81-29-838-6091; fax: +81-29-838-6091
E-mail: hsezutsu@affrc.go.jp

DOI: 10.1534/g3.114.011643

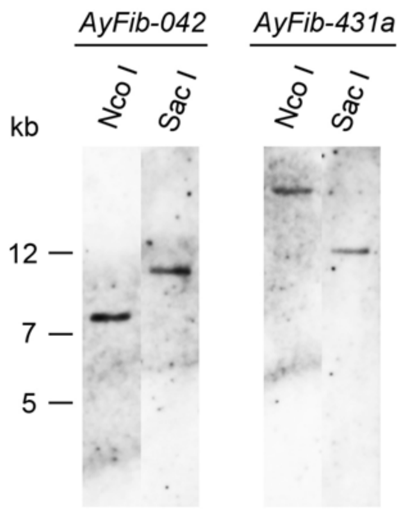

Figure S1 Southern hybridyzation of *AyFib-042* and *AyFib-431a* genomic DNAs. A single band was detected in both strains. Note the difference in band potisions in *AyFib-042* and *AyFib-431a*.

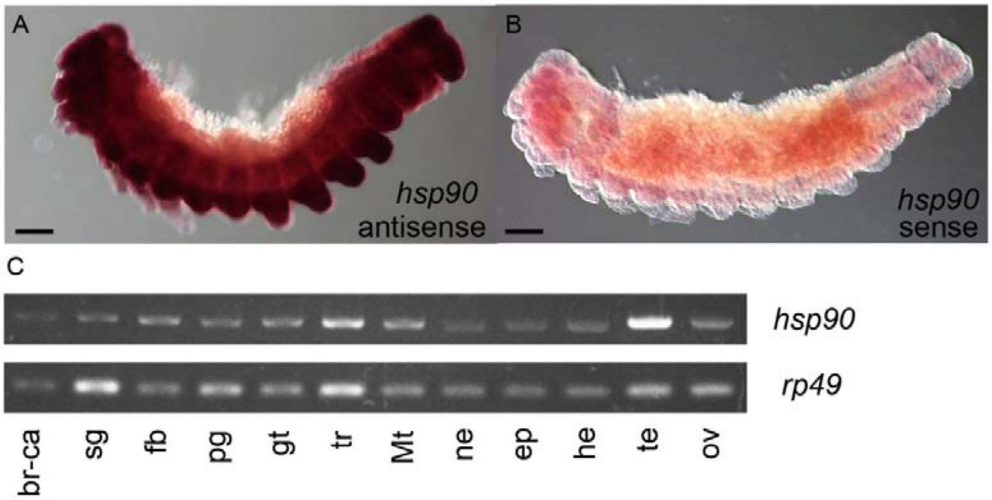

Figure S2 Expression of *hsp90*. (A, B) *In situ* hybridyzation of *hsp90* in a stage 20 embryo. The embryo was stained with an *hsp90* antisense (A) or sense (B) probe. Bar represents 0.1 mm. (C) RT-PCR of *hsp90* (upper panel) or *rp49* (lower panel) in each tissue of a *w-c* spinning stage larva. Abbreviations are: br-ca, brain-corpora allata; sg, silk gland; fb, fat body; pg, prothoracic gland; gt, gut; tr, trachea; Mt, Malpighian tubule; ne, ventral nerves; ep, epidermis; he, hemocyte; te, testis; ov, ovary.

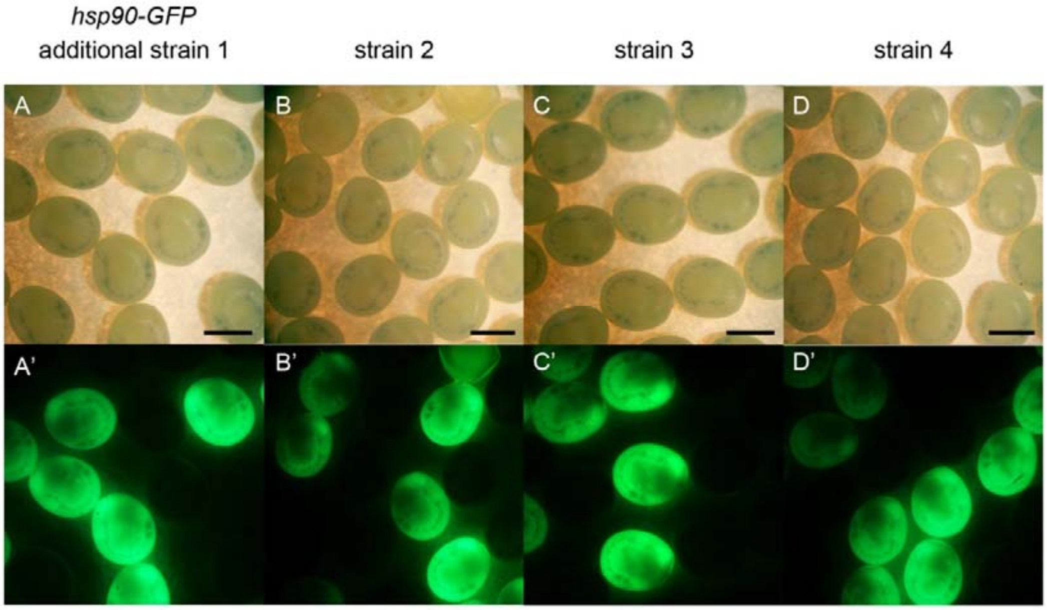

Figure S3 Embryonic GFP expression in four additional *hsp90-GFP* transgenic strains. (A, A') Strain 1. (B, B') Strain 2. (C, C') Strain 3. (D, D') Strain 4. (A, B, C, D) Bright-field images. (A', B', C', D') GFP images. Bar represents 1 mm.

1 CCATGGCTCAGTTCGCTTTAAATATCGATAGTCTTTAAAAAAAAATTATGATGTGAAATCTGAAAAGGTTTGCGCACTATGCGCTCATGC 90

91 AATTTCAACTTTAATCAATAGCCCTAAAGCAGTGCTGCCAACAAATAAAAAAATATTTATTGTAAGTGAGATAACTAAATTTGTCAGAAT 180

181 TCATCGCTTCTTTGCTATTTTTTATTATGGTTTGTGTTTAATTATATTTTAAACGTGTTGATGAAATAGTAAGATAATTAGCATTAAATT 270

271 TACACCACTTTCGTGAAGGTTTTCTGGCTAATCGTTTCAGTTTGTGTGGCATAAAACTATGGGCTATAACTATTGCTGGTGAGACTTTAA 360

361 CAGCTTCATAAGGACGGGTGGATTAACTCAAAAACACAGCTTGGAGGGAAAGAGTTTGCTAATAGCCGCCAGAGTGCCTCCAAAAGGGGT 450

451 TTAATAGTACTAGGACAGTTACTTTACGAGTGCATCTACTCCGTGTCGAGAAGCTTAGAGCTCAGTATCACATACAAATCGTCGACGAGT 540

541 TAGCTGAACCATGCTTTTATTGCTCTTGGCGTTAAAATTAGTAGTGATAATGAGAACGTATCAGGTGGATCTTGCATTATTTGAAAGATG 630

631 AGACTAAAGATCTGTAGGTTGTGCGAGAGAATGTCGCGAGGCAAAAATAGTTTCCTCTACCTGTAGATAACGAGAACACAAATTTTATAC 720

721 AGGGTGTATAGGAGAAGACGAGGAACAATTATTTTAATGATTTCTGACATCTAAATGAACAATAAGATTTTACTTTTGATGAACAAAACA 810

811 AGTAACTCACATCTTAAACTGAACGTTACTACGTTCTGGTTTGCTATTGTAATTTTAATCAATGTTTTCTCCAATCTGCATTATGATACA 900

901 ATAAAAGCTCATTAT*TCGCGTTTCCTTCACTCGCGTTTTCACTATTCGCAGATTAAAATGTGTCCCAATTCCTATTTTCGCAATTAAAGA* 990

991 *TCTCTTTATTCGCGGATCTAATCGATACATAGACATTGATAAAAAGAAATCCAATATTAAGTATTCAACTGAATATCCTTTATAATCGCG* 1080

1081 *CTATAATCACGGTTTATCTATAAAGCCTTGTTCGGACTAGGCGAGTATTTTAGTCGAGTACCGAGTAATTTAGTAGCTAAATGTGTCCGA* 1170

1171 *GCACCAAAAGTTTAGTCGAGTAGGTTAGTAAATAATTTAGTAAAGCCAATCCATTTTGTTCTATTTTATCGGGCGAGTAGCGCCTCCCAC* 1260

1261 *CACGCGTCCTAGCTCACTGGCTCACTGACTAAACAAGTCCGAACCTGTTGATTAGTCGAGTTCATTAGTGGCGCTTCTCAAATGGAACTT* 1350

1351 *TCGGACATTCTTAAACTATCGAAGTATTCTTTGGGATCTCTTCCAATGCTAATTCTTTCAGAAGACATTTATCTCTTCAATCGATCCATT* 1440

1441 *TTCGTACCTATAACTTATTTTTCATAATCACAGAAATTTGCTCGTCCACCTTTTCTAAAATAGCCATTACGATTAGCTTAATCAGTTTAT* 1530

1531 *TCACTCTATTGCGTTGCAGTCTGTTCATCCTGCAAAACACCACTCACTTGTGTACTCTACTGGACCGTAATGCGAACGATTTCTGTGCAG* 1620

1621 *TAGCGAGTAGTTTAGCCACTAAATTACTCGGTACTCGACTAAAATACTCGCCTAGTCCGAACAAGGCTTTAGCGACATATTTACGTAACG* 1710

1711 *TATACACCCCGATAAAGAGATTTACTGTCCAAAAAAAAACGTCAAAAAAATGGAACAAATTATGTGCTTTTTATTTATTGGTATTACTAATT* 1800

1801 *TCGTTAAGAATTTCATCGGAAGCAGCCAGCGCCGAATGCGGTGAATAGTAAAACTATAAGAAGGAAGCGGTTGGAATGATCTGGGTAAAA* 1890

1891 *TGTTATTTCTTACGAATTACGATGATTCTTTGATTAAATTTGGAAAAGTTTTGTGGTCAAATTATTTAACATTCATCTTGTATCATTTAT* 1980

1981 *TGACTCTAATTTCAACTAAGTTATTTCTGTAATGTTAAAATTTCGATGCTTTTCTAGAATATTCAGGAATGTTCCAGATATTCTTGTGTG* 2070

HSE-3 HSE-2

2071 *TAGATCGTATTGCCACCTTGTGCCAAATAGCGGCACTAAAATAATTCGGCAGAGCTATACAACATTTGGTAATTTTATTTATTTTGCTAT* 2160

2161 *TTTCCCCAGTTTTTTAAATTACTGTGTTTTTAACTAGCAATTACATTTAATTGGATGCAATTTACACTGCCTTAATAATTTTTTTTTAGT* 2250

2251 *TGTAATTTGGTTTCTTACGTTTATGCTGTATATTTTTAGTGATCTGCAAAATGCGAAACACTGAACAGCTTAGGAAAATTTAAAATGTGA* 2340

2341 *GATGTTTCTTTTACTTAAATAAATACTTAAAATATAACAGAGAAAAAACATTTTTAACTAGTCTGGCATATTTTAGTGAATCTATTGATG* 2430

2431 *AAAATGAACGAAATATGTAATTACCTTTATAATTTCTTGAATTATTTTAAGGAAATATATTTTTCCTAACACTTTAAACTTCAAAGCGGG* 2520

2521 *TGATGCAATAATAACTAACACAAAAAGAAACTTGCAGTGTTAAGTAAATGATTTTTCTCTTCGTTTTATGGCCTCCCAAAATGAAATAAT* 2610

2611 *TGCCAATTAATTTGAAATGATTAATTATTTTTATTTAATATTGTTTCGGTTTGTATTGATGGTAAAATGTTGATGCTCTCTGACGGAAAT* 2700

2701 *AGCGTCAGAATCGAGAAACTTCTACTGATTCATAGATGTCGCTTGCTAGAGGAAAGTATAAAAACGAATTTACACACCGCGCCCggcgtt* 2790

HSE-1

2791 *atttgaactaagagaagtacggagactaacgtttgatactttgcgctttgaaacacgtgtgttaaaaaccctctagtcattttgtgtgaa* 2880

2881 *ttaa* 2884

Figure S4 Sequence of *Bombyx* hsp90$^{P2.9k}$ fragment. The boxed region shows three putative HSE elements. The transcribed region of *hsp90* is written in lower case. The hsp90$^{P2.0k}$ sequence is shown in the italic character. The underlined region shows the genomic fragment used for *hsp90-GAL4* transgenic strain in Uchino *et al*. (2006).
