# Supplementary material for: Identification of a Novel Strong and Ubiquitous Promoter/Enhancer in the Silkworm Bombyx mori
Source: G3 (Bethesda). 2014 May 23;4(7):1347–57. doi: 10.1534/g3.114.011643 (PMC4455783; doi:10.1534/g3.114.011643)
Supplement: Supporting Information [file supp_g3.114.011643_FigureS1.pdf]

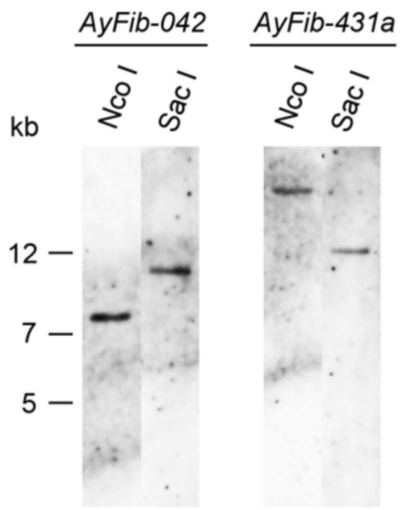

Figure S1 Southern hybridyzation of *AyFib-042* and *AyFib-431a* genomic DNAs. A single band was detected in both strains. Note the difference in band potisions in *AyFib-042* and *AyFib-431a*.
