# Supplementary material for: Identification of a Novel Strong and Ubiquitous Promoter/Enhancer in the Silkworm Bombyx mori
Source: G3 (Bethesda). 2014 May 23;4(7):1347–57. doi: 10.1534/g3.114.011643 (PMC4455783; doi:10.1534/g3.114.011643)
Supplement: Supporting Information [file supp_g3.114.011643_FigureS2.pdf]

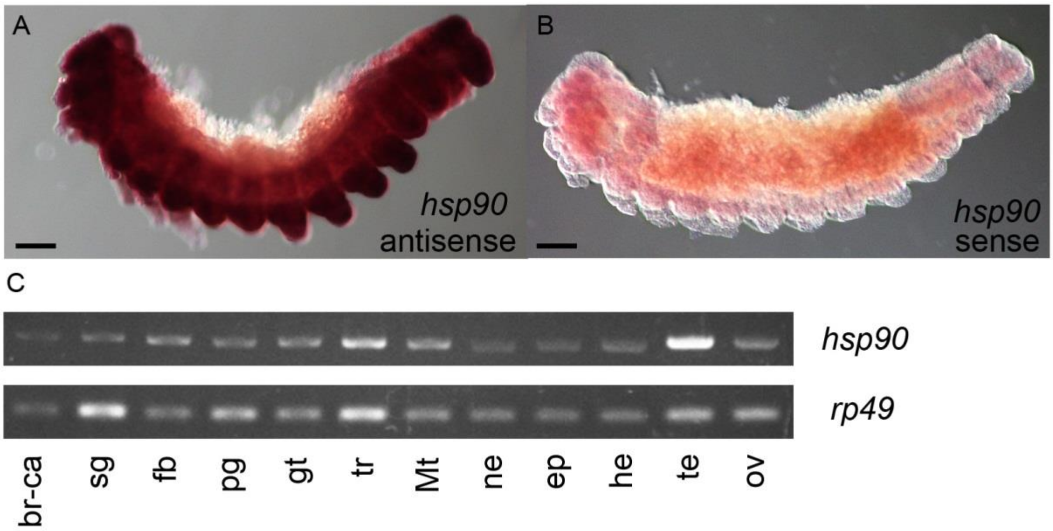

Figure S2 Expression of *hsp90*. (A, B) *In situ* hybridyzation of *hsp90* in a stage 20 embryo. The embryo was stained with an *hsp90* antisense (A) or sense (B) probe. Bar represents 0.1 mm. (C) RT-PCR of *hsp90* (upper panel) or *rp49* (lower panel) in each tissue of a *w-c* spinning stage larva. Abbreviations are: br-ca, brain-corpora allata; sg, silk gland; fb, fat body; pg, prothoracic gland; gt, gut; tr, trachea; Mt, Malpighian tubule; ne, ventral nerves; ep, epidermis; he, hemocyte; te, testis; ov, ovary.
