# Supplementary material for: Identification of a Novel Strong and Ubiquitous Promoter/Enhancer in the Silkworm Bombyx mori
Source: G3 (Bethesda). 2014 May 23;4(7):1347–57. doi: 10.1534/g3.114.011643 (PMC4455783; doi:10.1534/g3.114.011643)
Supplement: Supporting Information [file supp_g3.114.011643_FigureS3.pdf]

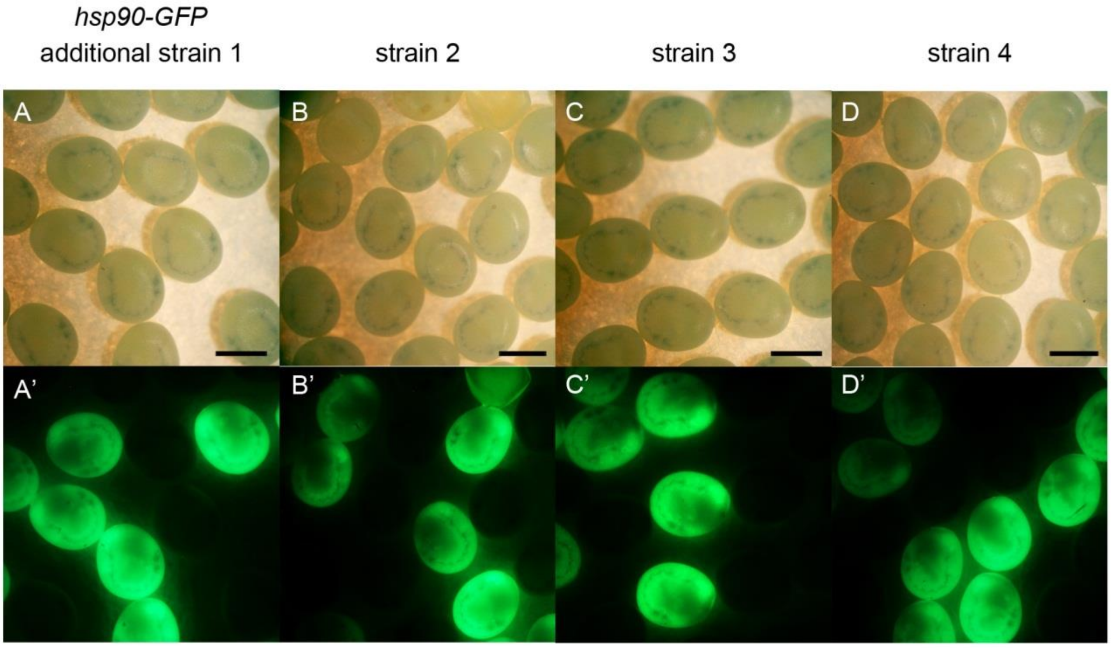

Figure S3 Embryonic GFP expression in four additional *hsp90-GFP* transgenic strains. (A, A') Strain 1. (B, B') Strain 2. (C, C') Strain 3. (D, D') Strain 4. (A, B, C, D) Bright-field images. (A', B', C', D') GFP images. Bar represents 1 mm.
