# Supplementary material for: Identification of a Novel Strong and Ubiquitous Promoter/Enhancer in the Silkworm Bombyx mori
Source: G3 (Bethesda). 2014 May 23;4(7):1347–57. doi: 10.1534/g3.114.011643 (PMC4455783; doi:10.1534/g3.114.011643)
Supplement: Supporting Information [file supp_g3.114.011643_FigureS4.pdf]

1 CCATGGCTCAGTTCGCTTTAAATATCGATAGTCTTTAAAAAAAAATTATGATGTGAAATCTGAAAAGGTTTGCGCACTATGCGCTCATGC 90

91 AATTTCAACTTTAATCAATAGCCCTAAAGCAGTGCTGCCAACAAATAAAAAAATATTTATTGTAAGTGAGATAACTAAATTTGTCAGAAT 180

181 TCATCGCTTCTTTGCTATTTTTTATTATGGTTTGTGTTTAATTATATTTTAAACGTGTTGATGAAATAGTAAGATAATTAGCATTAAATT 270

271 TACACCACTTTCGTGAAGGTTTTCTGGCTAATCGTTTCAGTTTGTGTGGCATAAAACTATGGGCTATAACTATTGCTGGTGAGACTTTAA 360

361 CAGCTTCATAAGGACGGGTGGATTAACTCAAAAACACAGCTTGGAGGGAAAGAGTTTGCTAATAGCCGCCAGAGTGCCTCCAAAAGGGGT 450

451 TTAATAGTACTAGGACAGTTACTTTACGAGTGCATCTACTCCGTGTCGAGAAGCTTAGAGCTCAGTATCACATACAAATCGTCGACGAGT 540

541 TAGCTGAACCATGCTTTTATTGCTCTTGGCGTTAAAATTAGTAGTGATAATGAGAACGTATCAGGTGGATCTTGCATTATTTGAAAGATG 630

631 AGACTAAAGATCTGTAGGTTGTGCGAGAGAATGTCGCGAGGCAAAAATAGTTTCCTCTACCTGTAGATAACGAGAACACAAATTTTATAC 720

721 AGGGTGTATAGGAGAAGACGAGGAACAATTATTTTAATGATTTCTGACATCTAAATGAACAATAAGATTTTACTTTTGATGAACAAAACA 810

811 AGTAACTCACATCTTAAACTGAACGTTACTACGTTCTGGTTTGCTATTGTAATTTTAATCAATGTTTTCTCCAATCTGCATTATGATACA 900

901 ATAAAAGCTCATTAT*TCGCGTTTCCTTCACTCGCGTTTTCACTATTCGCAGATTAAAATGTGTCCCAATTCCTATTTTCGCAATTAAAGA* 990

991 *TCTCTTTATTCGCGGATCTAATCGATACATAGACATTGATAAAAAGAAATCCAATATTAAGTATTCAACTGAATATCCTTTATAATCGCG* 1080

1081 *CTATAATCACGGTTTATCTATAAAGCCTTGTTCGGACTAGGCGAGTATTTTAGTCGAGTACCGAGTAATTTAGTAGCTAAATGTGTCCGA* 1170

1171 *GCACCAAAAGTTTAGTCGAGTAGGTTAGTAAATAATTTAGTAAAGCCAATCCATTTTGTTCTATTTTATCGGGCGAGTAGCGCCTCCCAC* 1260

1261 *CACGCGTCCTAGCTCACTGGCTCACTGACTAAACAAGTCCGAACCTGTTGATTAGTCGAGTTCATTAGTGGCGCTTCTCAAATGGAACTT* 1350

1351 *TCGGACATTCTTAAACTATCGAAGTATTCTTTGGGATCTCTTCCAATGCTAATTCTTTCAGAAGACATTTATCTCTTCAATCGATCCATT* 1440

1441 *TTCGTACCTATAACTTATTTTTCATAATCACAGAAATTTGCTCGTCCACCTTTTCTAAAATAGCCATTACGATTAGCTTAATCAGTTTAT* 1530

1531 *TCACTCTATTGCGTTGCAGTCTGTTCATCCTGCAAAACACCACTCACTTGTGTACTCTACTGGACCGTAATGCGAACGATTTCTGTGCAG* 1620

1621 *TAGCGAGTAGTTTAGCCACTAAATTACTCGGTACTCGACTAAAATACTCGCCTAGTCCGAACAAGGCTTTAGCGACATATTTACGTAACG* 1710

1711 *TATACACCCCGATAAAGAGATTTACTGTCCAAAAAAAAACGTCAAAAAATGGAACAAATTATGTGCTTTTTATTTATTGGTATTACTAATT* 1800

1801 *TCGTTAAGAATTTCATCGGAAGCAGCCAGCGCCGAATGCGGTGAATAGTAAAACTATAAGAAGGAAGCGGTTGGAATGATCTGGGTAAAA* 1890

1891 *TGTTATTTCTTACGAATTACGATGATTCTTTGATTAAATTTGGAAAAGTTTTGTGGTCAAATTATTTAACATTCATCTTGTATCATTTAT* 1980

1981 *TGACTCTAATTTCAACTAAGTTATTTCTGTAATGTTAAAATTTCGATGCTTTTCTAGAATATTCAGGAATGTTCCAGATATTCTTGTGTG* 2070

HSE-3 HSE-2

2071 *TAGATCGTATTGCCACCTTGTGCCAAATAGCGGCACTAAAATAATTCGGCAGAGCTATACAACATTTGGTAATTTTATTTATTTTGCTAT* 2160

2161 *TTTCCCCAGTTTTTTAAATTACTGTGTTTTTAACTAGCAATTACATTTAATTGGATGCAATTTACACTGCCTTAATAATTTTTTTTTAGT* 2250

2251 *TGTAATTTGGTTTCTTACGTTTATGCTGTATATTTTTAGTGATCTGCAAAATGCGAAACACTGAACAGCTTAGGAAAATTTAAAATGTGA* 2340

2341 *GATGTTTCTTTTACTTAAATAAATACTTAAAATATAACAGAGAAAAAACATTTTTAACTAGTCTGGCATATTTTAGTGAATCTATTGATG* 2430

2431 *AAAATGAACGAAATATGTAATTACCTTTATAATTTCTTGAATTATTTTAAGGAAATATATTTTTCCTAACACTTTAAACTTCAAAGCGGG* 2520

2521 *TGATGCAATAATAACTAACACAAAAAGAAACTTGCAGTGTTAAGTAAATGATTTTTCTCTTCGTTTTATGGCCTCCCAAAATGAAATAAT* 2610

2611 *TGCCAATTAATTTGAAATGATTAATTATTTTTATTTAATATTGTTTCGGTTTGTATTGATGGTAAAATGTTGATGCTCTCTGACGGAAAT* 2700

2701 *AGCGTCAGAATCGAGAAACTTCTACTGATTCATAGATGTCGCTTGCTAGAGGAAAGTATAAAAACGAATTTACACACCGCGCCCggcgtt* 2790

HSE-1

2791 *atttgaactaagagaagtacggagactaacgtttgatactttgcgctttgaaacacgtgtgttaaaaaccctctagtcattttgtgtgaa* 2880

2881 *ttaa* 2884

Figure S4 Sequence of *Bombyx* hsp90^P2.9k fragment. The boxed region shows three putative HSE elements. The transcribed region of *hsp90* is written in lower case. The hsp90^P2.0k sequence is shown in the italic character. The underlined region shows the genomic fragment used for *hsp90-GAL4* transgenic strain in Uchino *et al*. (2006).
